# Supplementary figures and images for: Comparative transcriptome analysis of roots, stems, and leaves of Pueraria lobata (Willd.) Ohwi: identification of genes involved in isoflavonoid biosynthesis
Source: PeerJ. 2021 Feb 22;9:e10885. doi: 10.7717/peerj.10885 (PMC7906042; doi:10.7717/peerj.10885)

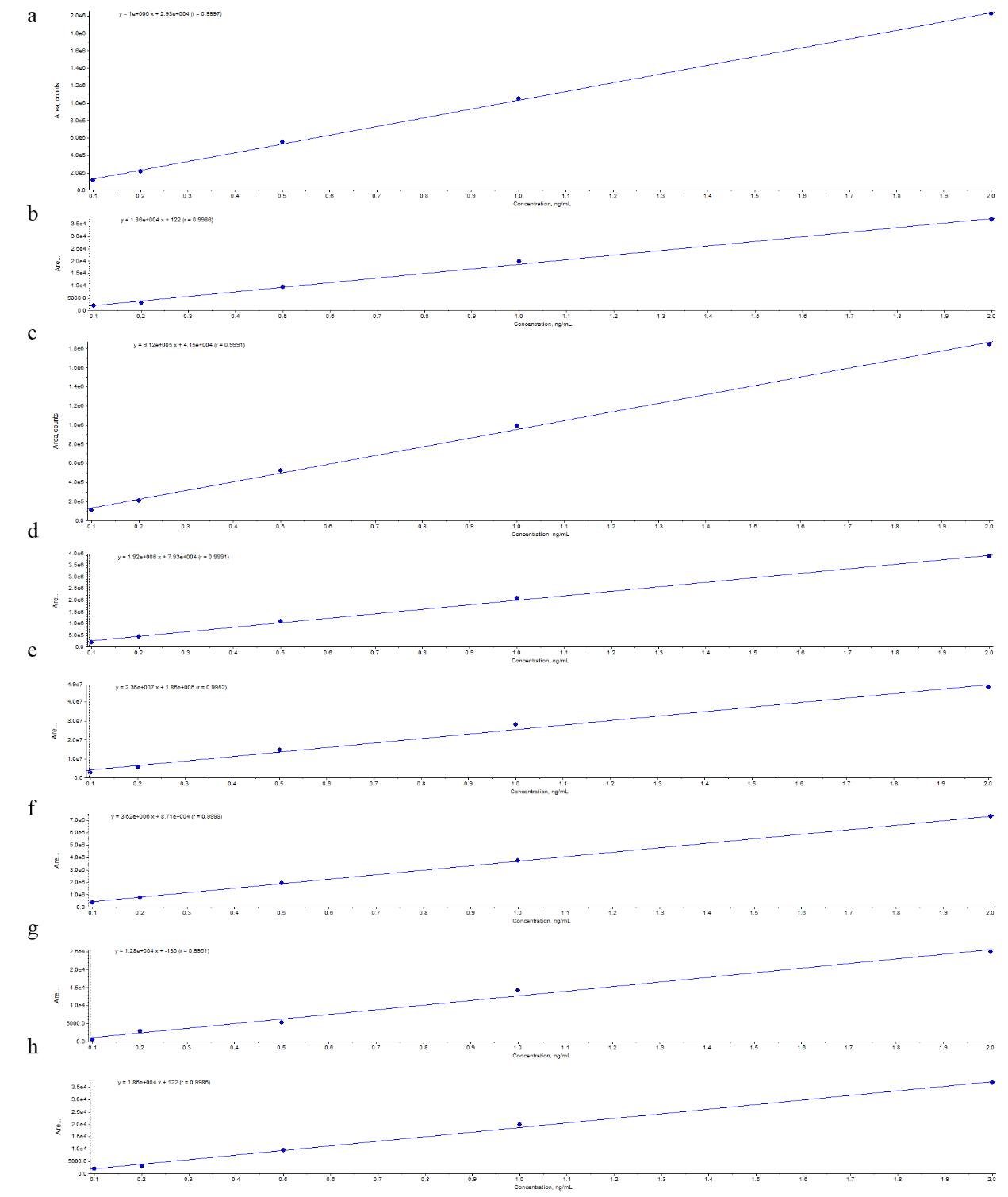

Supplement: Supplemental Information 1 — a, b, c, d, e, f, g, h is genistin, glycitin, daidzein, genistein, formononetin, puerarin, daidzin, and glycitein, respectively. [file peerj-09-10885-s001.png]

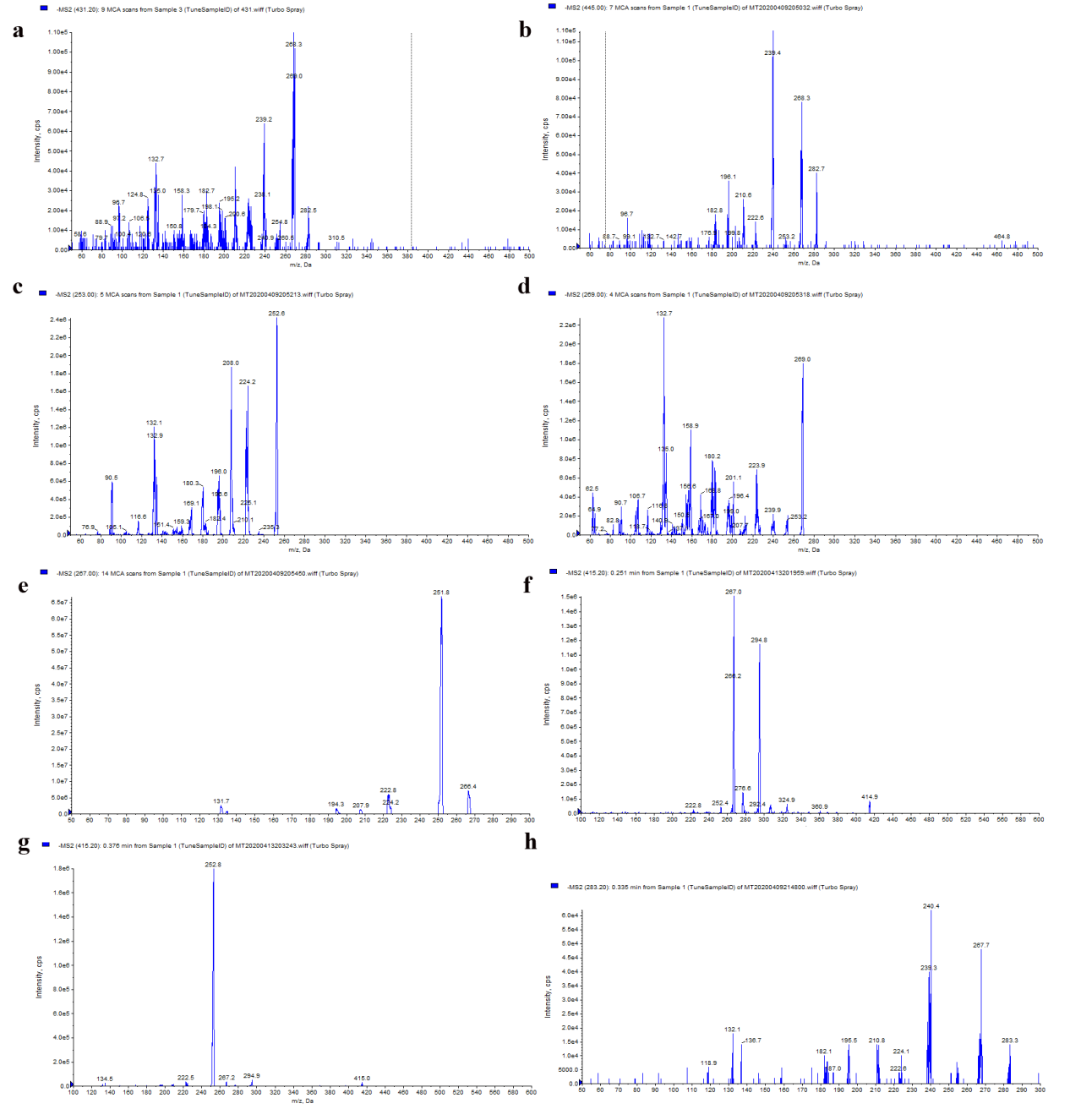

Supplement: Supplemental Information 2 — a, b, c, d, e, f, g, h is genistin, glycitin, daidzein, genistein, formononetin, puerarin, daidzin, and glycitein of P. lobata by HPLC-MS/MS in negative ion mode. [file peerj-09-10885-s002.png]

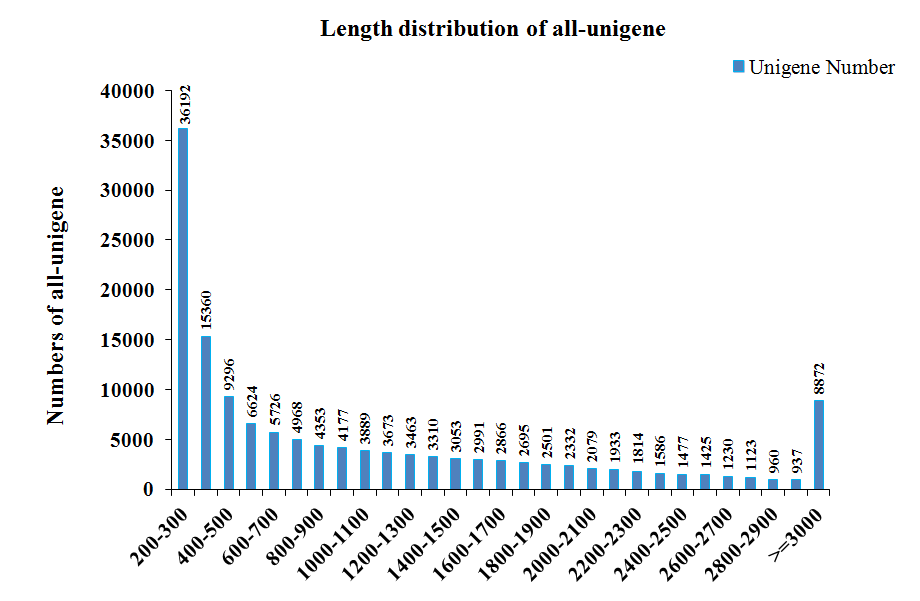

Supplement: Supplemental Information 3 [file peerj-09-10885-s003.png]

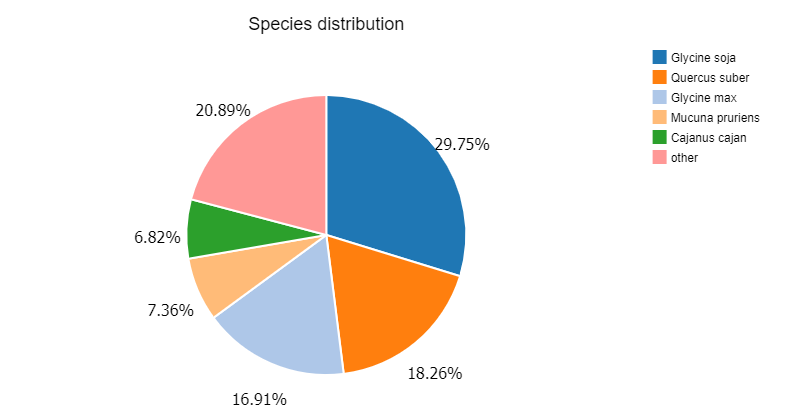

Supplement: Supplemental Information 4 [file peerj-09-10885-s004.png]

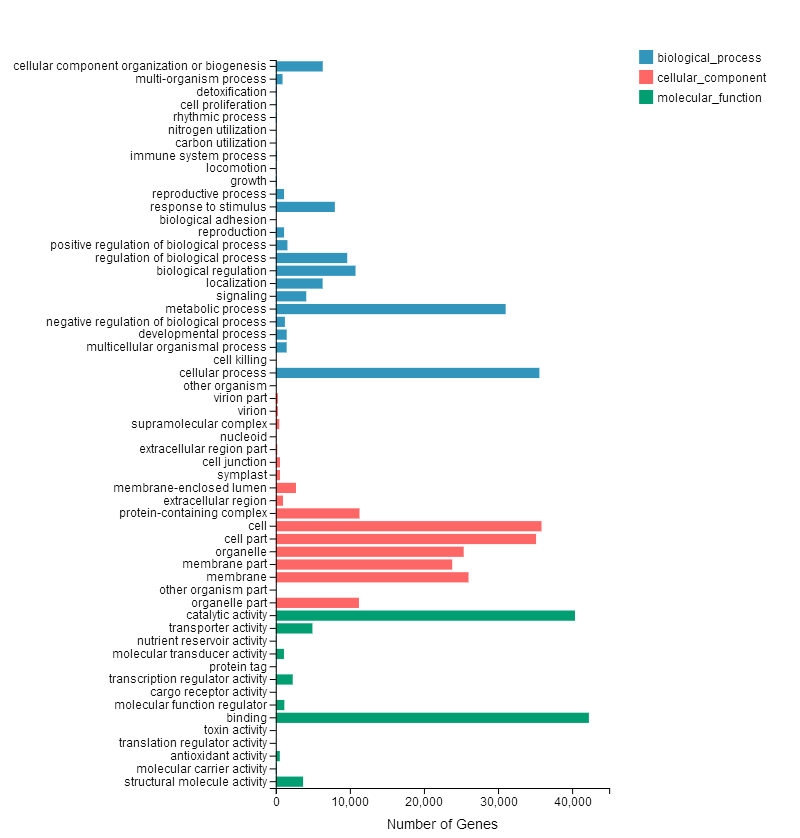

Supplement: Supplemental Information 5 [file peerj-09-10885-s005.png]

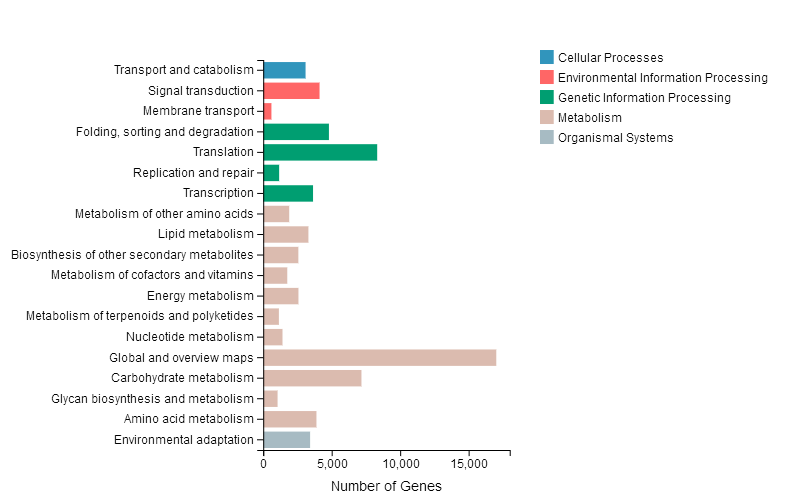

Supplement: Supplemental Information 6 [file peerj-09-10885-s006.png]

### Actin

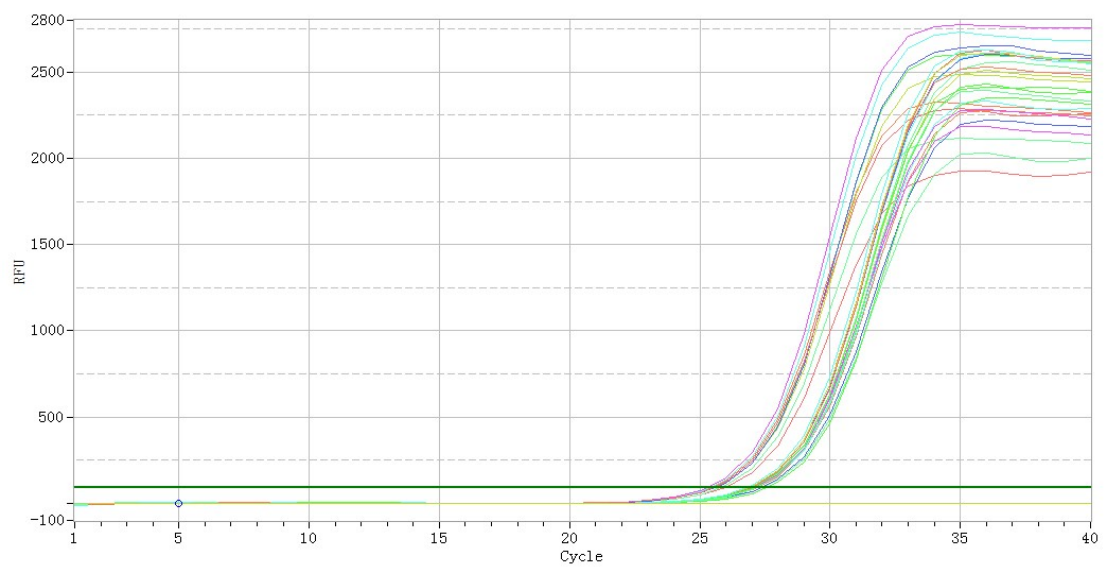

### CL518.Contig3

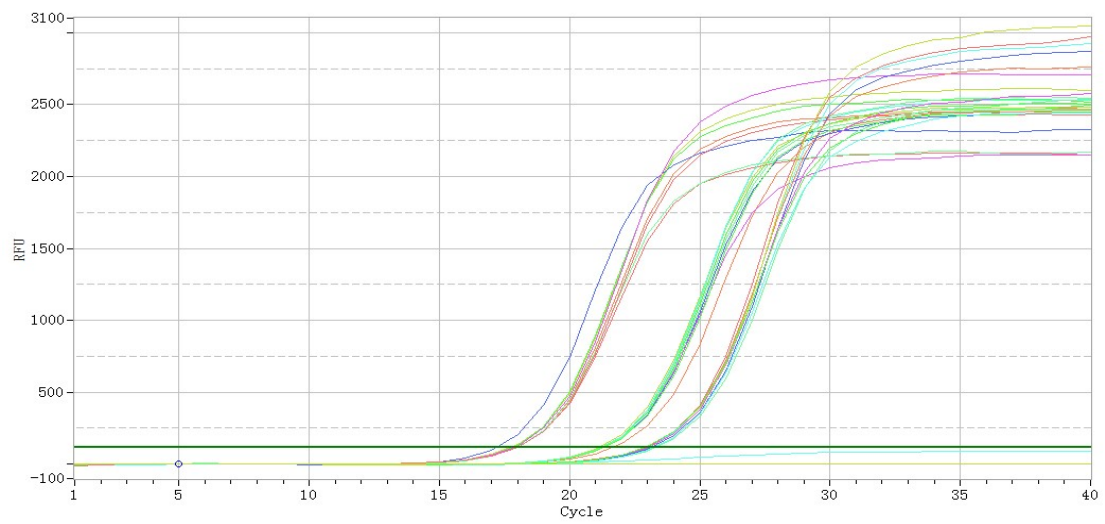

### CL1520.Contig3

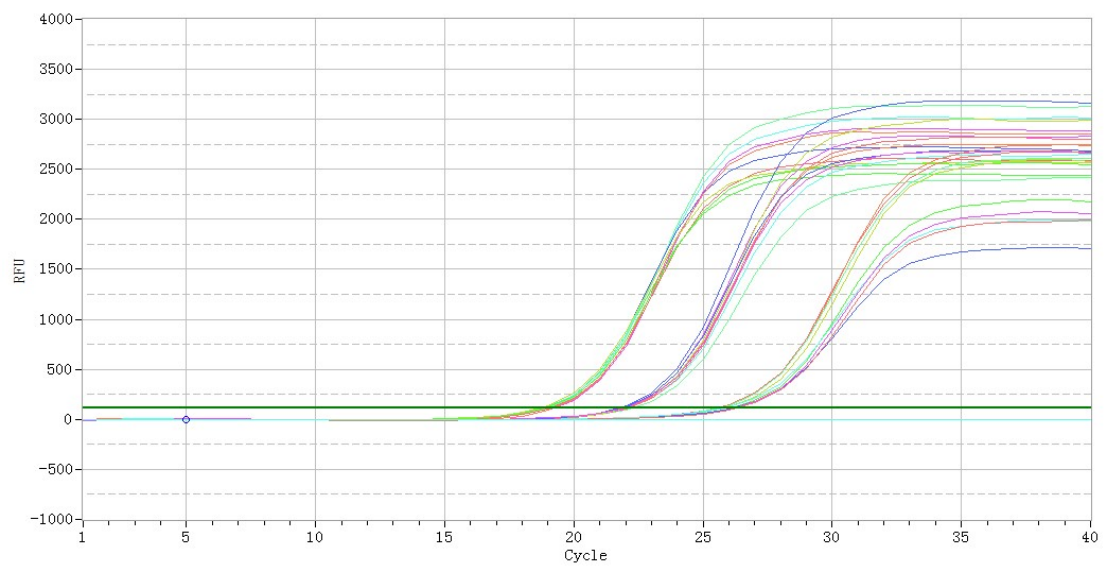

CL2444.Contig2

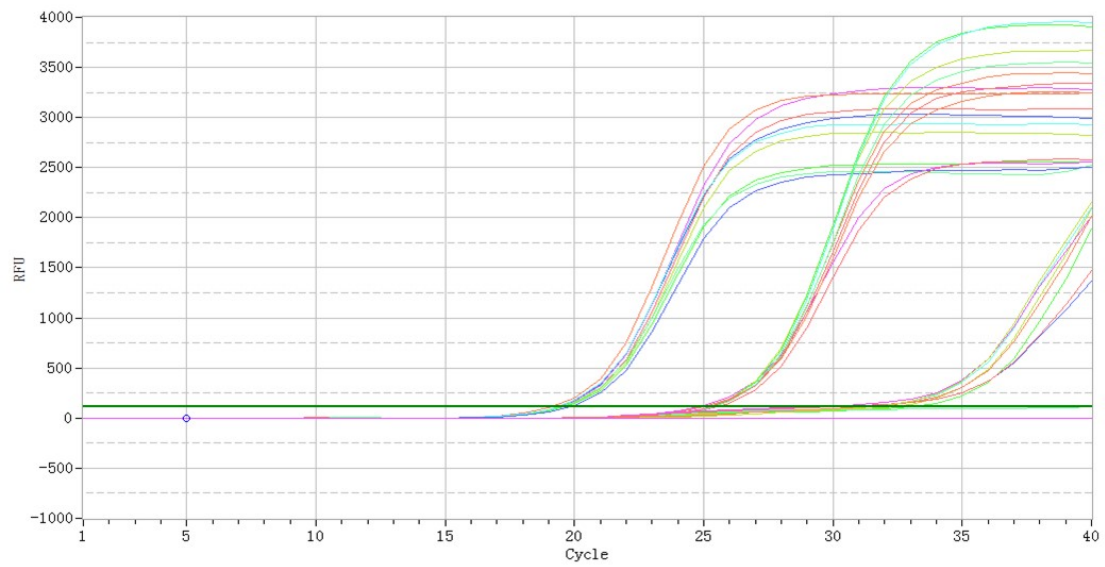

CL2625.Contig2

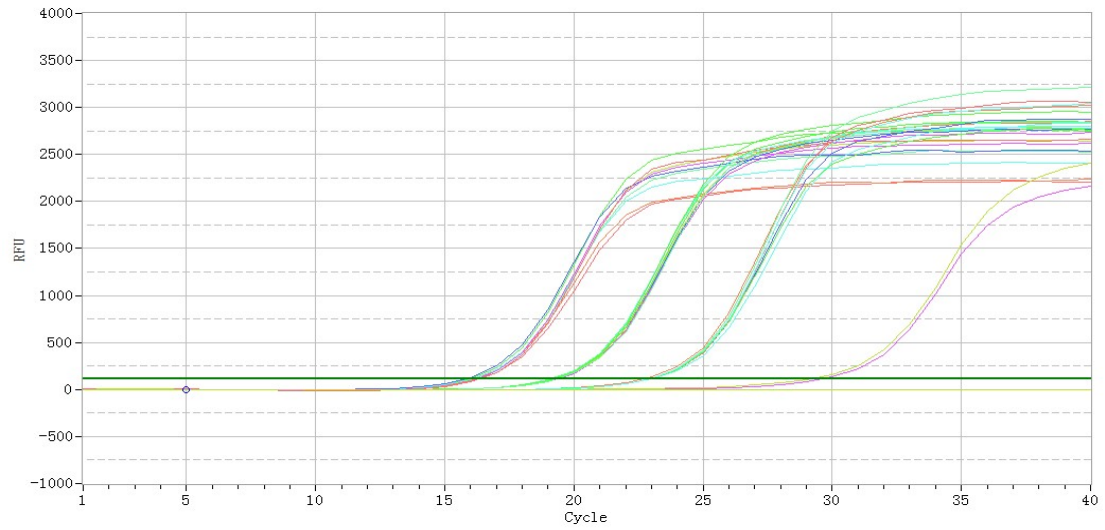

CL3338.Contig1

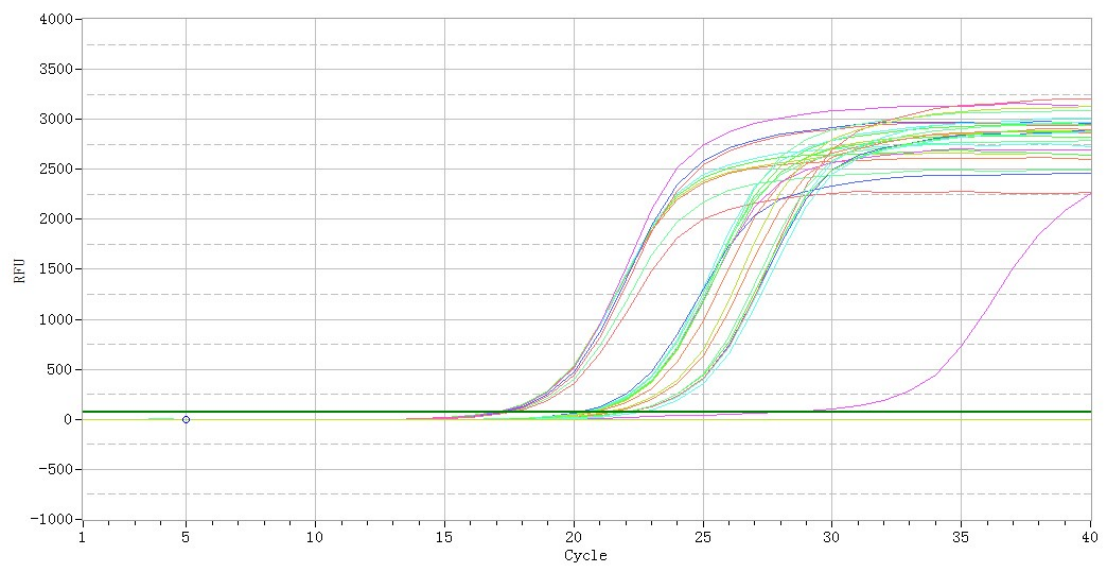

CL10538.Contig1

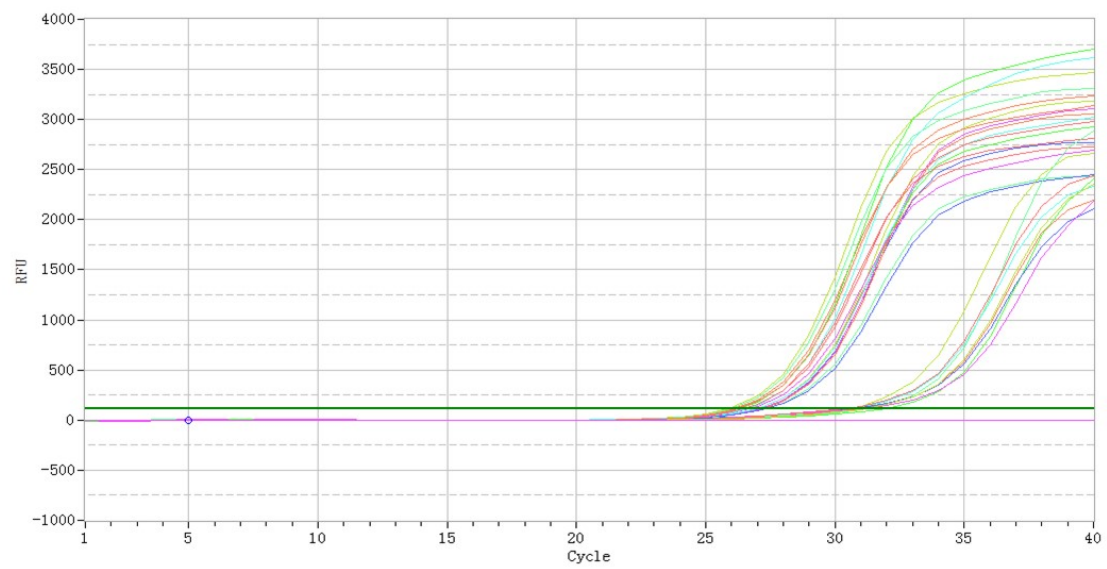

Unigene9945

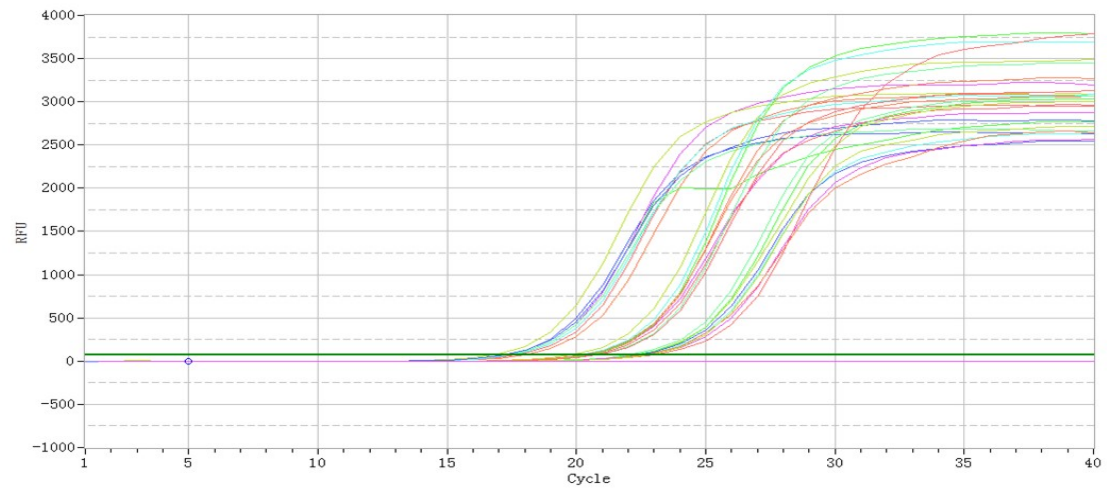

Unigene54862

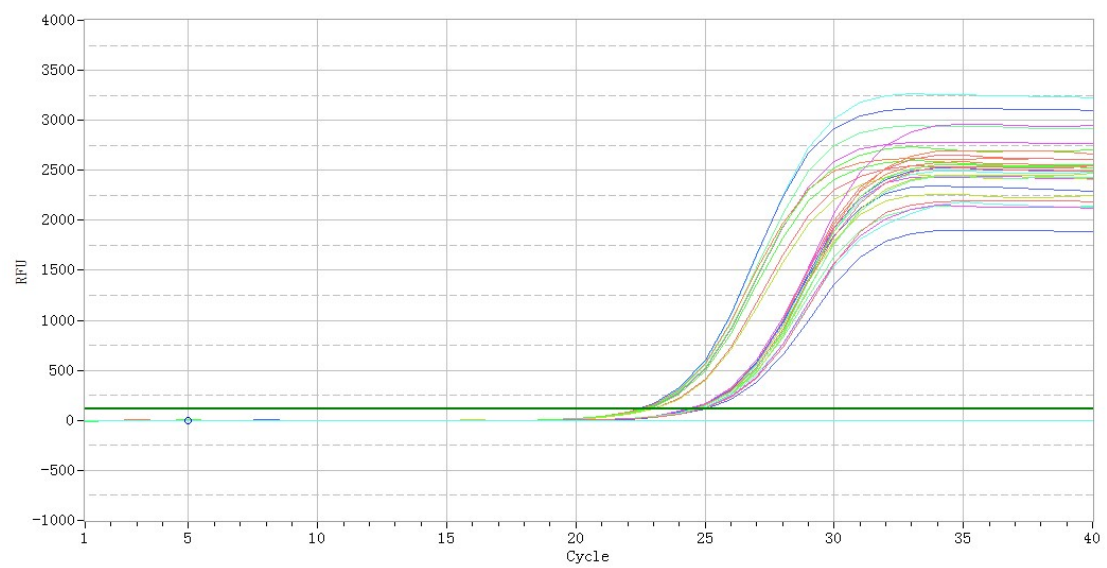

# Unigene72602

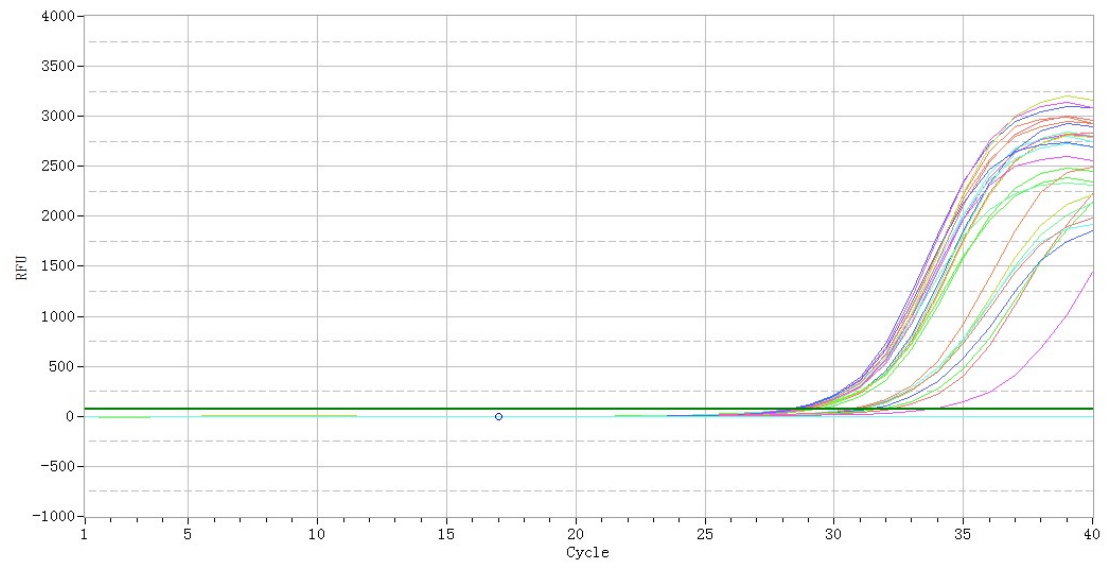

Supplement: Supplemental Information 21 [file peerj-09-10885-s021.pdf]

### Actin

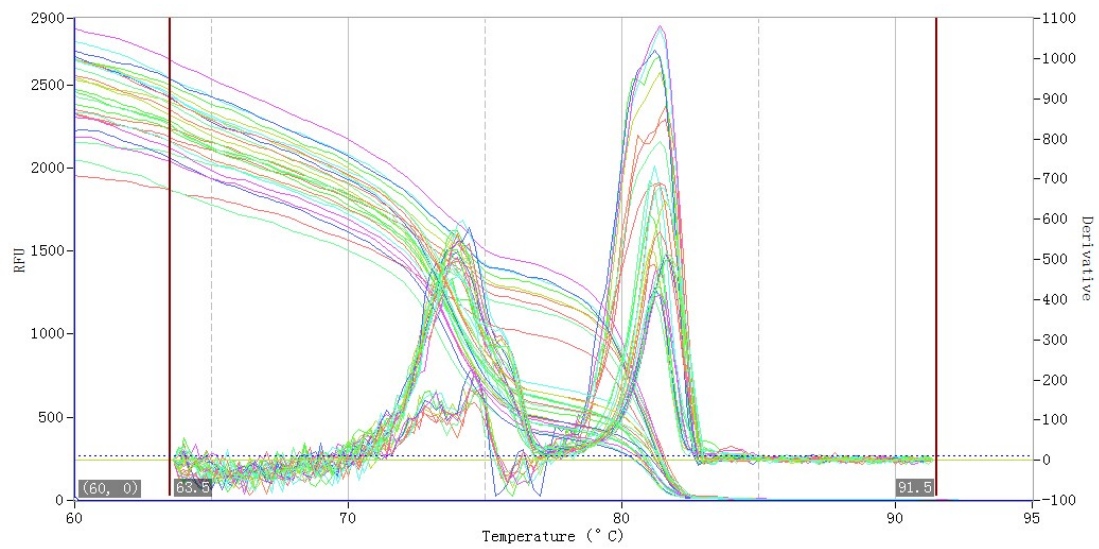

### CL518.Contig3

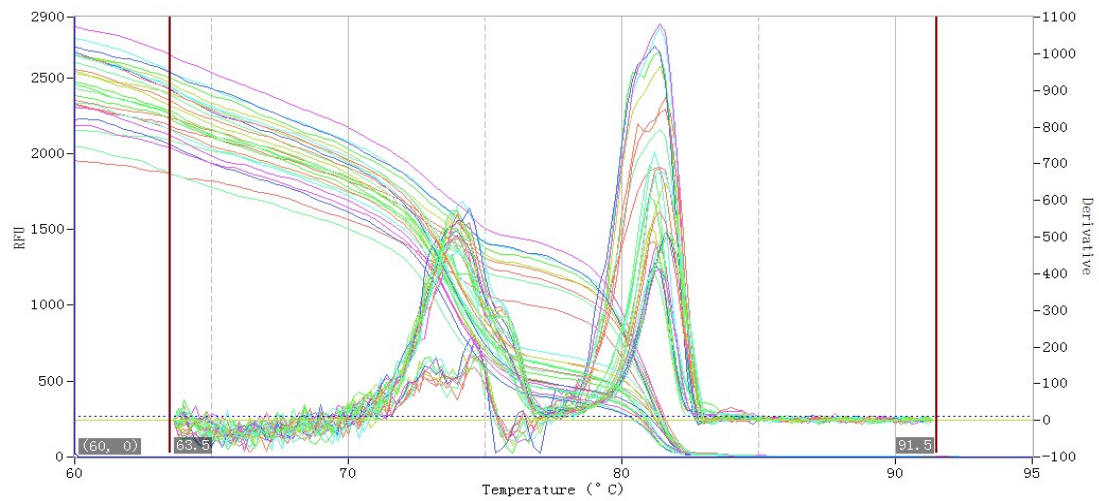

### CL1520.Contig3

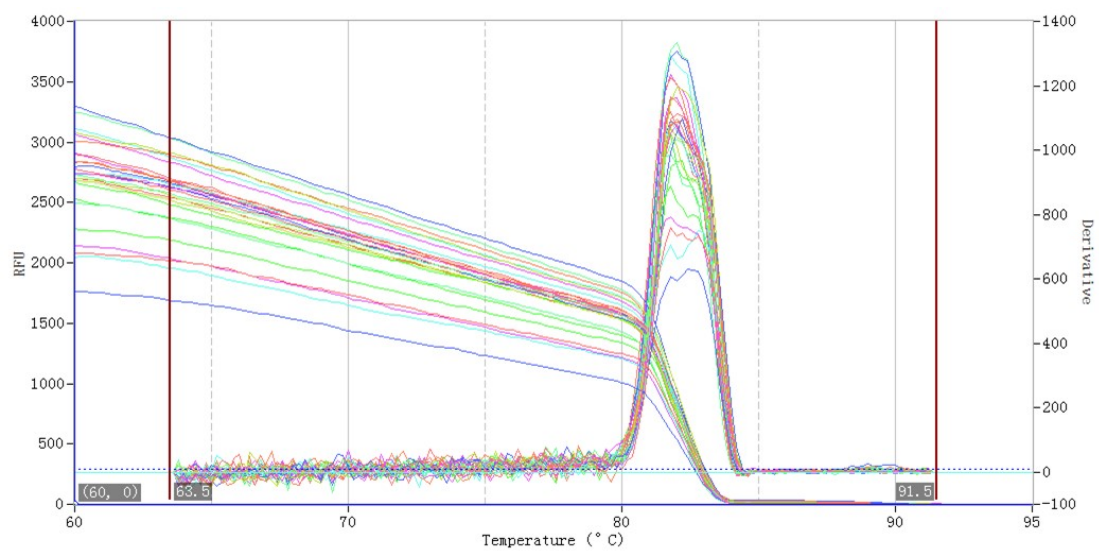

CL2444.Contig2

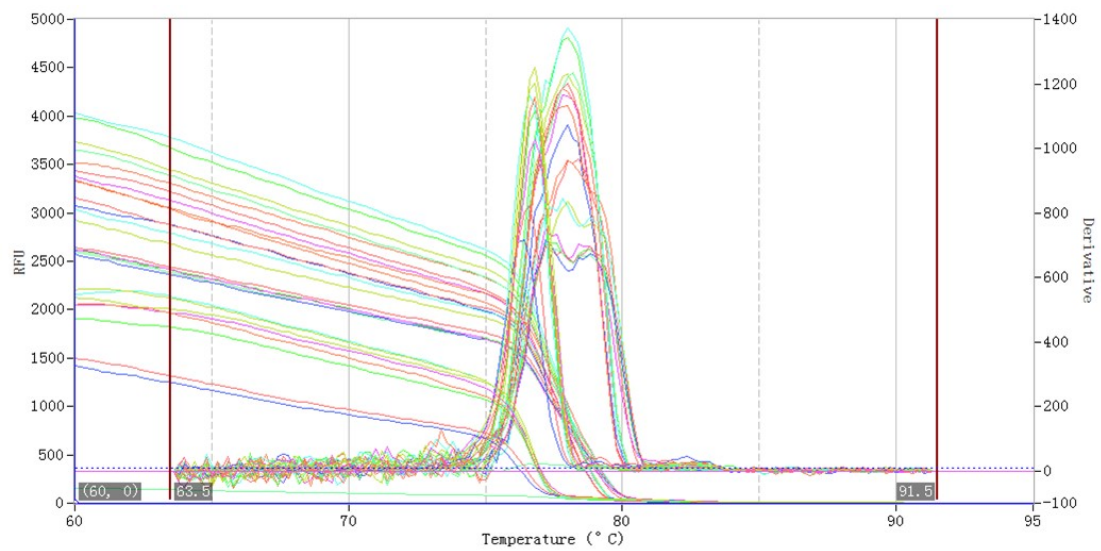

CL2625.Contig2

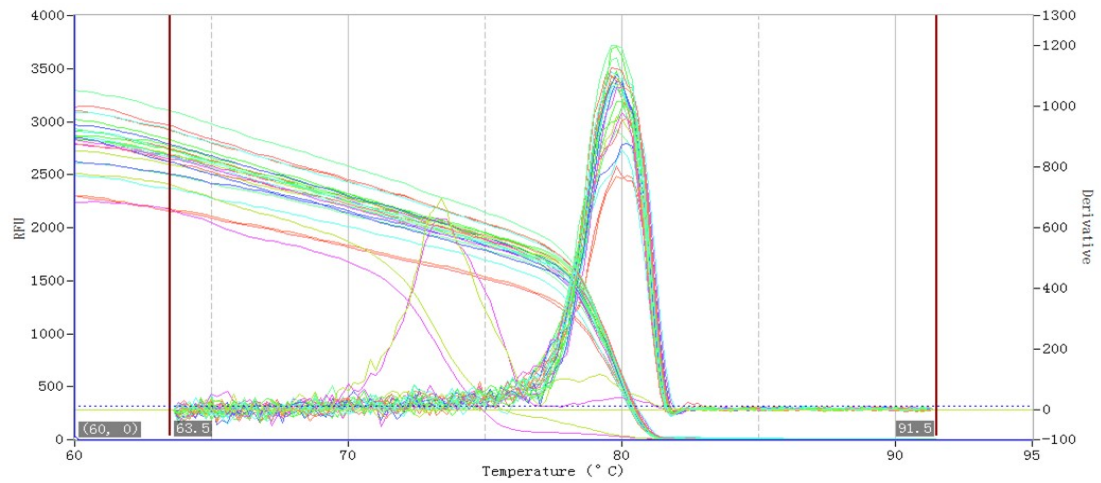

CL3338.Contig1

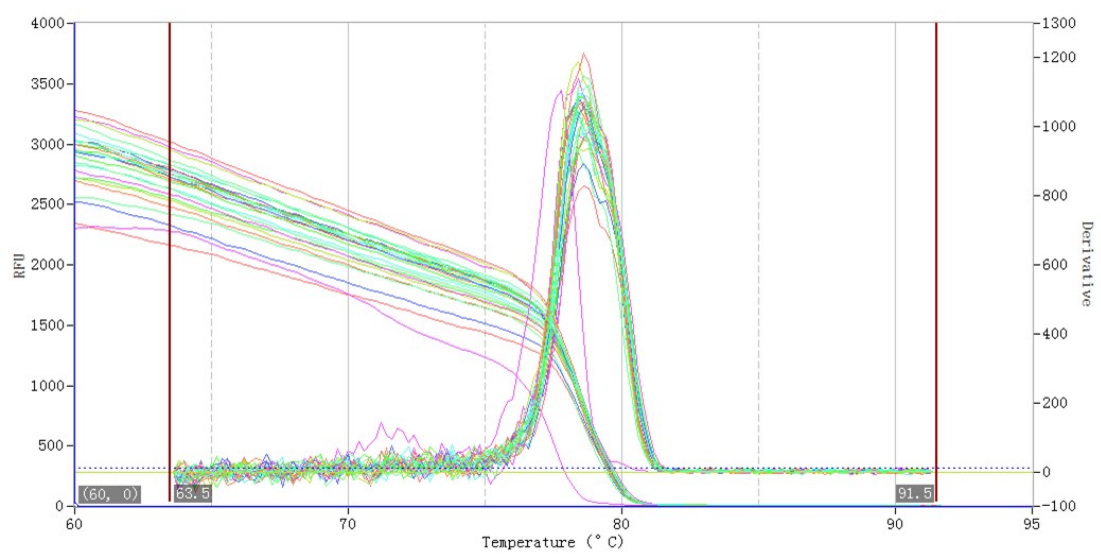

CL10538.Contig1

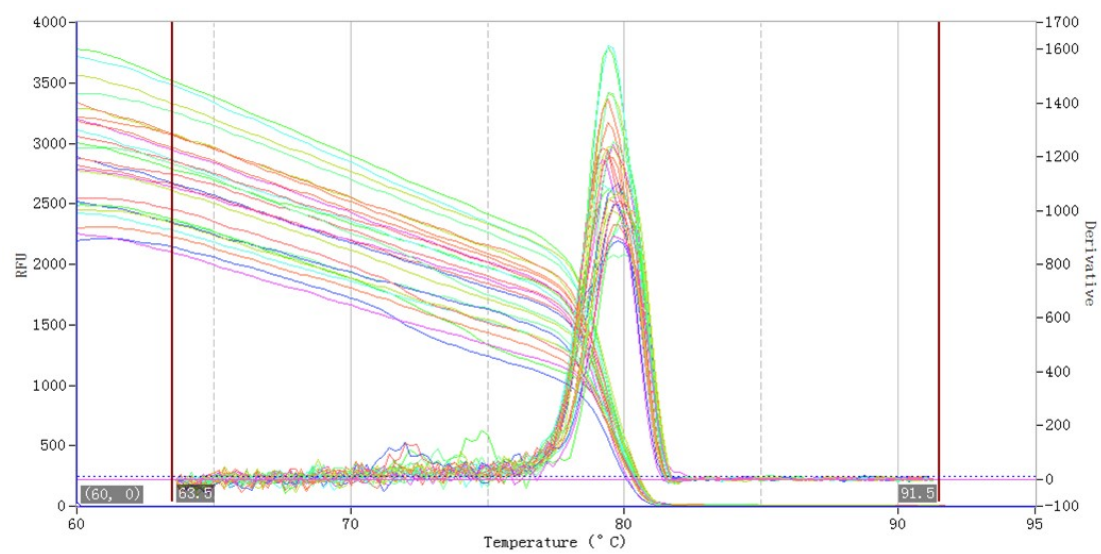

Unigene9945

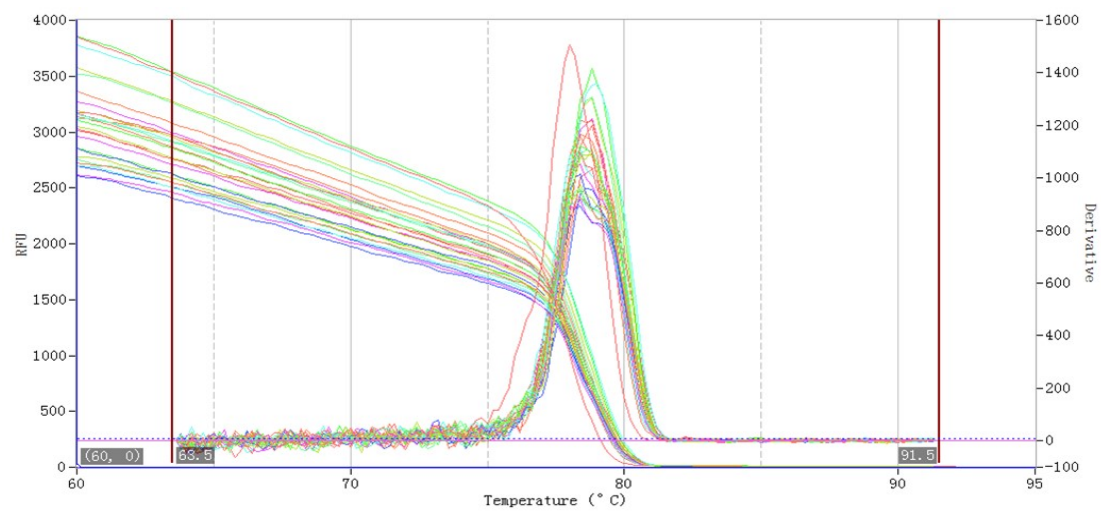

Unigene54862

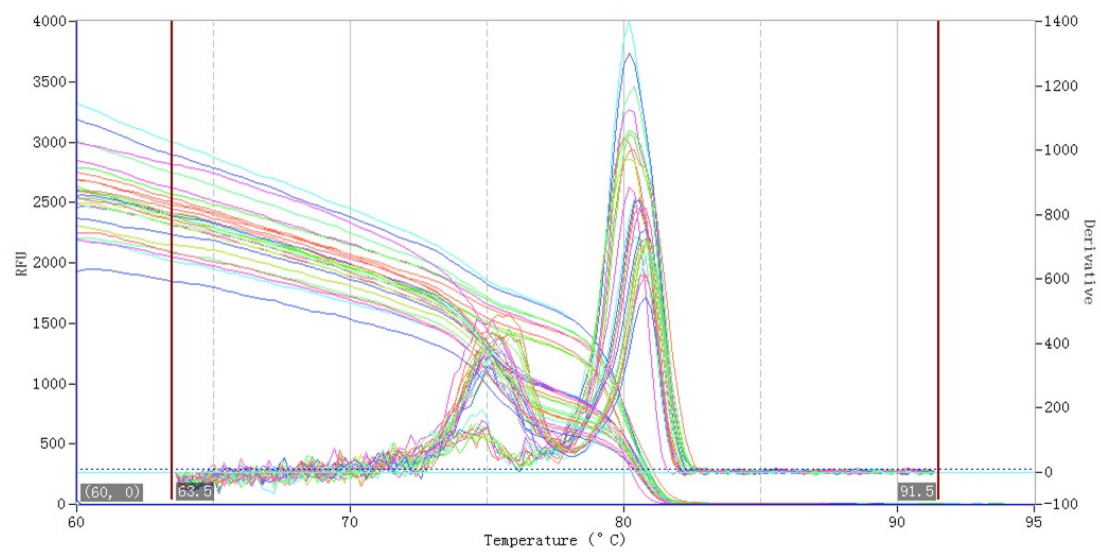

## Unigene72602

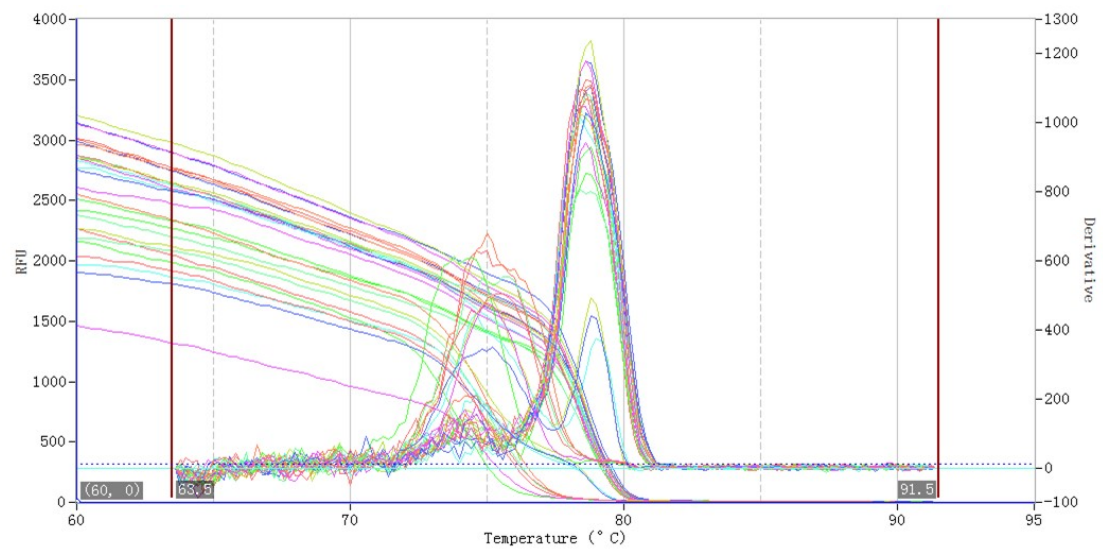

Supplement: Supplemental Information 22 [file peerj-09-10885-s022.pdf]
