# Supplementary material for: Comparative transcriptome analysis of roots, stems, and leaves of Pueraria lobata (Willd.) Ohwi: identification of genes involved in isoflavonoid biosynthesis
Source: PeerJ. 2021 Feb 22;9:e10885. doi: 10.7717/peerj.10885 (PMC7906042; doi:10.7717/peerj.10885)
Supplement: Supplemental Information 7 [file peerj-09-10885-s007.docx]

**Supplementary Table S1.** RNA information of different tissues.

| Sample | Concentration (ng/μL) | Total amount (μg) | OD260/280 | RIN | 28S/18S |
| --- | --- | --- | --- | --- | --- |
| Leaves | 516.6 | 10.33 | 1.56 | 7.1 | 1.1 |
| Roots | 713.7 | 14.27 | 1.97 | 7.8 | 1.8 |
| Stems | 317.8 | 6.36 | 2.00 | 7.3 | 1.0 |
