# Supplementary material for: Comparative transcriptome analysis of roots, stems, and leaves of Pueraria lobata (Willd.) Ohwi: identification of genes involved in isoflavonoid biosynthesis
Source: PeerJ. 2021 Feb 22;9:e10885. doi: 10.7717/peerj.10885 (PMC7906042; doi:10.7717/peerj.10885)
Supplement: Supplemental Information 8 [file peerj-09-10885-s008.docx]

**Supplementary Table S2.** The optimized MS parameters of isofavonoid compounds.

| **Analyte** | **Parent ion**（m/z） | **Product ion**（m/z） | **Retention time (ms)** | **Tube lens**（V） | **Collision energy**（V） |
| --- | --- | --- | --- | --- | --- |
| glycitein | 283 | 240 | 30 | 100 | 34 |
|  |  | 268 | 30 |  | 23 |
| formononetin | 267 | 252 | 30 | 100 | 30 |
|  |  | 223 | 30 |  | 40 |
| genistein | 269 | 224 | 30 | 100 | 37 |
|  |  | 183 | 30 |  | 35 |
| daidzein | 253 | 224 | 30 | 130 | 35 |
|  |  | 208 | 30 |  | 40 |
| genistin | 431 | 268 | 30 | 140 | 39 |
|  |  | 239 | 30 |  | 60 |
| glycitin | 445 | 268 | 30 | 90 | 42 |
|  |  | 283 | 30 |  | 20 |
| puerarin | 415 | 295 | 30 | 80 | 32 |
| daidzin | 415 | 253 | 30 | 80 | 44 |
