# Supplementary material for: Comparative transcriptome analysis of roots, stems, and leaves of Pueraria lobata (Willd.) Ohwi: identification of genes involved in isoflavonoid biosynthesis
Source: PeerJ. 2021 Feb 22;9:e10885. doi: 10.7717/peerj.10885 (PMC7906042; doi:10.7717/peerj.10885)
Supplement: Supplemental Information 9 [file peerj-09-10885-s009.docx]

**Supplementary Table S3.** qRT-PCR Primers used in this study.

| **Enzyme** | **Unigene** | **Amplicon Size（bp）** | **Forward primer（5'→3'）** | **Reverse primer（5'→3'）** |
| --- | --- | --- | --- | --- |
| PAL | CL518.Contig3 | 135 | TGGCTTCTATTGTGCTCTTTG | CAGGGTGGTGCTTTAGTTTATG |
| C4H | CL2444.Contig2 | 121 | GAGTGAGGAGCGAAGGGTTA | GCCTGAATGAACAAAGGGTC |
| 4CL | CL1520.Contig3 | 163 | ACGGGATGACTGAAGCAGGAC | TCACCGGGTTGATTGTAGCC |
| CHS | CL3338.Contig1 | 88 | CCAGACAGTGAAGGGGCTAT | TTGAGACAATCCCAGGAACA |
| CHI | Unigene9945 | 172 | TCCAGCATCCATCACTAACG | GGCCTTGTCTTCCAAATAAAC |
| F6H | Unigene72602 | 84 | GAGCACGCACCATCAGAAAC | GGGTACAAATCTCCCAAGTCAA |
| IFS2 | CL2625.Contig2 | 155 | ACCATTGGAATTTCGTCCTG | TTGCCATTCCTGAAGTAGCC |
| HIDH | CL10538.Contig1 | 152 | TCTCATCCAAAGACATCGTCA | GTGGTAGAGTTGGGAGAAAGC |
| IF7GT | Unigene54862 | 86 | ATTGGACCCGTGATTTCTTC | ACGCTATGACTCGGTTGTGA |
