# Supplementary material for: Comparative transcriptome analysis of roots, stems, and leaves of Pueraria lobata (Willd.) Ohwi: identification of genes involved in isoflavonoid biosynthesis
Source: PeerJ. 2021 Feb 22;9:e10885. doi: 10.7717/peerj.10885 (PMC7906042; doi:10.7717/peerj.10885)
Supplement: Supplemental Information 10 [file peerj-09-10885-s010.docx]

**Supplementary Table S4.** Statistics of transcript quality.

| **Sample** | **Total Raw Reads (M)** | **Total Clean Reads (M)** | **Total Clean Bases (Gb)** | **Clean Reads Q30(%)** | **Clean** Reads Ratio (%) |
| --- | --- | --- | --- | --- | --- |
| Leaf | 78.88 | 71.07 | 10.66 | 89.75 | 90.1 |
| Stem | 78.88 | 69.28 | 10.39 | 89.34 | 87.83 |
| Root | 78.88 | 70.56 | 10.58 | 89.25 | 89.45 |
