# Supplementary material for: Comparative transcriptome analysis of roots, stems, and leaves of Pueraria lobata (Willd.) Ohwi: identification of genes involved in isoflavonoid biosynthesis
Source: PeerJ. 2021 Feb 22;9:e10885. doi: 10.7717/peerj.10885 (PMC7906042; doi:10.7717/peerj.10885)
Supplement: Supplemental Information 11 [file peerj-09-10885-s011.docx]

**Supplementary Table S5.** Statistics of unigene information.

| **Sample** | **Total Number** | **Total**  **Length** | **Mean Length** | **N50** | **N70** | **N90** | **GC (%)** |
| --- | --- | --- | --- | --- | --- | --- | --- |
| Leaf | 70522 | 80060373 | 1135 | 1769 | 1216 | 524 | 40.36 |
| Stem | 101048 | 102501263 | 1014 | 1724 | 1091 | 399 | 42.03 |
| Root | 83080 | 91420364 | 1100 | 1818 | 1209 | 461 | 41.36 |
| All-Unigene | 140905 | 152641311 | 1083 | 1883 | 1215 | 428 | 41.64 |
