# Supplementary material for: Comparative transcriptome analysis of roots, stems, and leaves of Pueraria lobata (Willd.) Ohwi: identification of genes involved in isoflavonoid biosynthesis
Source: PeerJ. 2021 Feb 22;9:e10885. doi: 10.7717/peerj.10885 (PMC7906042; doi:10.7717/peerj.10885)
Supplement: Supplemental Information 12 [file peerj-09-10885-s012.docx]

**Supplementary Table S6.** KEGG pathway annotation.

| Pathway ID |  | Pathway Name | Gene Number |
| --- | --- | --- | --- |
| ko00010 |  | Glycolysis / Gluconeogenesis | 1207 |
| ko00020 |  | Citrate cycle (TCA cycle) | 808 |
| ko00030 |  | Pentose phosphate pathway | 471 |
| ko00040 |  | Pentose and glucuronate interconversions | 1047 |
| ko00051 |  | Fructose and mannose metabolism | 438 |
| ko00052 |  | Galactose metabolism | 790 |
| ko00053 |  | Ascorbate and aldarate metabolism | 511 |
| ko00061 |  | Fatty acid biosynthesis | 387 |
| ko00062 |  | Fatty acid elongation | 192 |
| ko00071 |  | Fatty acid degradation | 544 |
| ko00072 |  | Synthesis and degradation of ketone bodies | 94 |
| ko00073 |  | Cutin, suberine and wax biosynthesis | 125 |
| ko00100 |  | Steroid biosynthesis | 241 |
| ko00130 |  | Ubiquinone and other terpenoid-quinone biosynthesis | 318 |
| ko00190 |  | Oxidative phosphorylation | 1173 |
| ko00195 |  | Photosynthesis | 150 |
| ko00196 |  | Photosynthesis - antenna proteins | 35 |
| ko00220 |  | Arginine biosynthesis | 341 |
| ko00230 |  | Purine metabolism | 949 |
| ko00232 |  | Caffeine metabolism | 29 |
| ko00240 |  | Pyrimidine metabolism | 669 |
| ko00250 |  | Alanine, aspartate and glutamate metabolism | 638 |
| ko00260 |  | Glycine, serine and threonine metabolism | 498 |
| ko00261 |  | Monobactam biosynthesis | 93 |
| ko00270 |  | Cysteine and methionine metabolism | 898 |
| ko00280 |  | Valine, leucine and isoleucine degradation | 577 |
| ko00290 |  | Valine, leucine and isoleucine biosynthesis | 220 |
| ko00300 |  | Lysine biosynthesis | 78 |
| ko00310 |  | Lysine degradation | 389 |
| ko00330 |  | Arginine and proline metabolism | 582 |
| ko00332 |  | Carbapenem biosynthesis | 8 |
| ko00340 |  | Histidine metabolism | 179 |
| ko00350 |  | Tyrosine metabolism | 418 |
| ko00360 |  | Phenylalanine metabolism | 395 |
| ko00380 |  | Tryptophan metabolism | 403 |
| ko00400 |  | Phenylalanine, tyrosine and tryptophan biosynthesis | 332 |
| ko00402 |  | Benzoxazinoid biosynthesis | 78 |
| ko00410 |  | beta-Alanine metabolism | 395 |
| ko00430 |  | Taurine and hypotaurine metabolism | 83 |
| ko00440 |  | Phosphonate and phosphinate metabolism | 79 |
| ko00450 |  | Selenocompound metabolism | 233 |
| ko00460 |  | Cyanoamino acid metabolism | 605 |
| ko00472 |  | D-Arginine and D-ornithine metabolism | 1 |
| ko00480 |  | Glutathione metabolism | 594 |
| ko00500 |  | Starch and sucrose metabolism | 1486 |
| ko00510 |  | N-Glycan biosynthesis | 306 |
| ko00511 |  | Other glycan degradation | 319 |
| ko00514 |  | Other types of O-glycan biosynthesis | 149 |
| ko00515 |  | Mannose type O-glycan biosynthesis | 8 |
| ko00520 |  | Amino sugar and nucleotide sugar metabolism | 1182 |
| ko00531 |  | Glycosaminoglycan degradation | 209 |
| ko00561 |  | Glycerolipid metabolism | 617 |
| ko00562 |  | Inositol phosphate metabolism | 637 |
| ko00563 |  | Glycosylphosphatidylinositol (GPI)-anchor biosynthesis | 152 |
| ko00564 |  | Glycerophospholipid metabolism | 668 |
| ko00565 |  | Ether lipid metabolism | 209 |
| ko00590 |  | Arachidonic acid metabolism | 178 |
| ko00591 |  | Linoleic acid metabolism | 132 |
| ko00592 |  | alpha-Linolenic acid metabolism | 459 |
| ko00600 |  | Sphingolipid metabolism | 420 |
| ko00601 |  | Glycosphingolipid biosynthesis - lacto and neolacto series | 5 |
| ko00603 |  | Glycosphingolipid biosynthesis - globo and isoglobo series | 64 |
| ko00604 |  | Glycosphingolipid biosynthesis - ganglio series | 140 |
| ko00620 |  | Pyruvate metabolism | 902 |
| ko00630 |  | Glyoxylate and dicarboxylate metabolism | 793 |
| ko00640 |  | Propanoate metabolism | 384 |
| ko00650 |  | Butanoate metabolism | 223 |
| ko00660 |  | C5-Branched dibasic acid metabolism | 76 |
| ko00670 |  | One carbon pool by folate | 144 |
| ko00710 |  | Carbon fixation in photosynthetic organisms | 602 |
| ko00730 |  | Thiamine metabolism | 142 |
| ko00740 |  | Riboflavin metabolism | 98 |
| ko00750 |  | Vitamin B6 metabolism | 121 |
| ko00760 |  | Nicotinate and nicotinamide metabolism | 211 |
| ko00770 |  | Pantothenate and CoA biosynthesis | 243 |
| ko00780 |  | Biotin metabolism | 91 |
| ko00785 |  | Lipoic acid metabolism | 22 |
| ko00790 |  | Folate biosynthesis | 160 |
| ko00860 |  | Porphyrin and chlorophyll metabolism | 278 |
| ko00900 |  | Terpenoid backbone biosynthesis | 355 |
| ko00901 |  | Indole alkaloid biosynthesis | 67 |
| ko00902 |  | Monoterpenoid biosynthesis | 43 |
| ko00903 |  | Limonene and pinene degradation | 96 |
| ko00904 |  | Diterpenoid biosynthesis | 186 |
| ko00905 |  | Brassinosteroid biosynthesis | 35 |
| ko00906 |  | Carotenoid biosynthesis | 262 |
| ko00908 |  | Zeatin biosynthesis | 86 |
| ko00909 |  | Sesquiterpenoid and triterpenoid biosynthesis | 128 |
| ko00910 |  | Nitrogen metabolism | 368 |
| ko00920 |  | Sulfur metabolism | 279 |
| ko00940 |  | Phenylpropanoid biosynthesis | 1590 |
| ko00941 |  | Flavonoid biosynthesis | 314 |
| ko00942 |  | Anthocyanin biosynthesis | 40 |
| ko00943 |  | Isoflavonoid biosynthesis | 166 |
| ko00944 |  | Flavone and flavonol biosynthesis | 106 |
| ko00945 |  | Stilbenoid, diarylheptanoid and gingerol biosynthesis | 87 |
| ko00950 |  | Isoquinoline alkaloid biosynthesis | 248 |
| ko00960 |  | Tropane, piperidine and pyridine alkaloid biosynthesis | 201 |
| ko00965 |  | Betalain biosynthesis | 125 |
| ko00966 |  | Glucosinolate biosynthesis | 32 |
| ko00970 |  | Aminoacyl-tRNA biosynthesis | 522 |
| ko00999 |  | Biosynthesis of secondary metabolites - unclassified | 5 |
| ko01040 |  | Biosynthesis of unsaturated fatty acids | 288 |
| ko01200 |  | Carbon metabolism | 2378 |
| ko01210 |  | 2-Oxocarboxylic acid metabolism | 721 |
| ko01212 |  | Fatty acid metabolism | 735 |
| ko01230 |  | Biosynthesis of amino acids | 2283 |
| ko02010 |  | ABC transporters | 600 |
| ko03008 |  | Ribosome biogenesis in eukaryotes | 1071 |
| ko03010 |  | Ribosome | 3502 |
| ko03013 |  | RNA transport | 2740 |
| ko03015 |  | mRNA surveillance pathway | 1423 |
| ko03018 |  | RNA degradation | 1205 |
| ko03020 |  | RNA polymerase | 1100 |
| ko03022 |  | Basal transcription factors | 394 |
| ko03030 |  | DNA replication | 578 |
| ko03040 |  | Spliceosome | 2182 |
| ko03050 |  | Proteasome | 334 |
| ko03060 |  | Protein export | 341 |
| ko03410 |  | Base excision repair | 243 |
| ko03420 |  | Nucleotide excision repair | 674 |
| ko03430 |  | Mismatch repair | 517 |
| ko03440 |  | Homologous recombination | 550 |
| ko03450 |  | Non-homologous end-joining | 41 |
| ko04016 |  | MAPK signaling pathway - plant | 2231 |
| ko04070 |  | Phosphatidylinositol signaling system | 594 |
| ko04075 |  | Plant hormone signal transduction | 1929 |
| ko04120 |  | Ubiquitin mediated proteolysis | 876 |
| ko04122 |  | Sulfur relay system | 82 |
| ko04130 |  | SNARE interactions in vesicular transport | 176 |
| ko04136 |  | Autophagy - other | 217 |
| ko04141 |  | Protein processing in endoplasmic reticulum | 2205 |
| ko04144 |  | Endocytosis | 1736 |
| ko04145 |  | Phagosome | 650 |
| ko04146 |  | Peroxisome | 625 |
| ko04626 |  | Plant-pathogen interaction | 2905 |
| ko04712 |  | Circadian rhythm - plant | 522 |
