# Supplementary material for: Comparative transcriptome analysis of roots, stems, and leaves of Pueraria lobata (Willd.) Ohwi: identification of genes involved in isoflavonoid biosynthesis
Source: PeerJ. 2021 Feb 22;9:e10885. doi: 10.7717/peerj.10885 (PMC7906042; doi:10.7717/peerj.10885)
Supplement: Supplemental Information 16 [file peerj-09-10885-s016.docx]

**Supplementary Table S7.** Predicted UGTs in *Pueraria lobata* (Wild.) Ohwi plant.

| **Query ID** | **Subject ID** | **Identity** | **Subject Annotation** |
| --- | --- | --- | --- |
| CL10082.Contig2 | XP_003531287.1 | 94.25 | flavonol 7-O-beta-glucosyltransferase UGT74F1 [Glycine max] |
| CL10210.Contig1 | XP_003538437.1 | 91.77 | UDP-glycosyltransferase 76B1 [Glycine max] |
| CL10210.Contig2 | XP_003538437.1 | 91.77 | UDP-glycosyltransferase 76B1 [Glycine max] |
| CL10210.Contig4 | XP_003538437.1 | 91.77 | UDP-glycosyltransferase 76B1 [Glycine max] |
| CL10210.Contig5 | XP_003538437.1 | 91.77 | UDP-glycosyltransferase 76B1 [Glycine max] |
| CL10817.Contig1 | ADV71369.1 | 99.37 | glycosyltransferase GT14A05 [Pueraria montana var. lobata] |
| CL11049.Contig1 | XP_006603934.1 | 87.42 | 7-deoxyloganetin glucosyltransferase [Glycine max] |
| CL11835.Contig1 | KHN39543.1 | 87.8 | UDP-glycosyltransferase 92A1 [Glycine soja] |
| CL12276.Contig2 | KHN24339.1 | 92.18 | UDP-glycosyltransferase 85A2 [Glycine soja] |
| CL12276.Contig4 | KHN24339.1 | 89.21 | UDP-glycosyltransferase 85A2 [Glycine soja] |
| CL12276.Contig5 | KHN24339.1 | 92.18 | UDP-glycosyltransferase 85A2 [Glycine soja] |
| CL12481.Contig2 | KHN15047.1 | 89.02 | Putative glycosyltransferase [Glycine soja] |
| CL12659.Contig1 | XP_003517884.1 | 82.66 | UDP-glycosyltransferase 89A2 [Glycine max] |
| CL12659.Contig2 | XP_003517884.1 | 82.66 | UDP-glycosyltransferase 89A2 [Glycine max] |
| CL12929.Contig1 | KHN13565.1 | 91.26 | UDP-glycosyltransferase 83A1 [Glycine soja] |
| CL1442.Contig10 | XP_015935152.1 | 79.22 | probable xyloglucan glycosyltransferase 12 [Arachis duranensis] |
| CL1442.Contig11 | XP_003544500.1 | 95.6 | probable xyloglucan glycosyltransferase 12 [Glycine max] |
| CL1442.Contig13 | KHN17187.1 | 95.27 | Putative xyloglucan glycosyltransferase 12 [Glycine soja] |
| CL1442.Contig14 | XP_003524038.1 | 95.93 | probable xyloglucan glycosyltransferase 12 [Glycine max] |
| CL1442.Contig1 | XP_003524038.1 | 95.93 | probable xyloglucan glycosyltransferase 12 [Glycine max] |
| CL1442.Contig2 | KHN17187.1 | 95.27 | Putative xyloglucan glycosyltransferase 12 [Glycine soja] |
| CL1442.Contig4 | XP_015935152.1 | 79.22 | probable xyloglucan glycosyltransferase 12 [Arachis duranensis] |
| CL1442.Contig5 | XP_003544500.1 | 95.6 | probable xyloglucan glycosyltransferase 12 [Glycine max] |
| CL1442.Contig7 | XP_015935152.1 | 78.42 | probable xyloglucan glycosyltransferase 12 [Arachis duranensis] |
| CL1442.Contig9 | XP_015935152.1 | 78.42 | probable xyloglucan glycosyltransferase 12 [Arachis duranensis] |
| CL1750.Contig11 | XP_020207781.1 | 79.18 | UDP-glycosyltransferase 79B30-like [Cajanus cajan] |
| CL1750.Contig12 | AMQ26123.1 | 97.6 | UDP-glycosyltransferase 6 [Pueraria montana var. lobata] |
| CL1750.Contig15 | XP_020207781.1 | 76.2 | UDP-glycosyltransferase 79B30-like [Cajanus cajan] |
| CL1750.Contig2 | XP_020207781.1 | 78.09 | UDP-glycosyltransferase 79B30-like [Cajanus cajan] |
| CL1750.Contig3 | XP_020207781.1 | 78.09 | UDP-glycosyltransferase 79B30-like [Cajanus cajan] |
| CL1750.Contig6 | AMQ26123.1 | 93.2 | UDP-glycosyltransferase 6 [Pueraria montana var. lobata] |
| CL1750.Contig8 | XP_020237137.1 | 87.22 | UDP-glycosyltransferase 79B30-like [Cajanus cajan] |
| CL2163.Contig1 | AMQ26117.1 | 99.18 | UDP-glycosyltransferase 47 [Pueraria montana var. lobata] |
| CL2163.Contig2 | AMQ26117.1 | 98.98 | UDP-glycosyltransferase 47 [Pueraria montana var. lobata] |
| CL2163.Contig4 | AMQ26117.1 | 99.63 | UDP-glycosyltransferase 47 [Pueraria montana var. lobata] |
| CL2269.Contig1 | AMQ26131.1 | 90.32 | UDP-glycosyltransferase 25 [Pueraria montana var. lobata] |
| CL2269.Contig2 | AMQ26131.1 | 90.32 | UDP-glycosyltransferase 25 [Pueraria montana var. lobata] |
| CL2269.Contig4 | AMQ26131.1 | 93.98 | UDP-glycosyltransferase 25 [Pueraria montana var. lobata] |
| CL2269.Contig5 | AMQ26131.1 | 96.77 | UDP-glycosyltransferase 25 [Pueraria montana var. lobata] |
| CL2269.Contig6 | AMQ26131.1 | 96.77 | UDP-glycosyltransferase 25 [Pueraria montana var. lobata] |
| CL2269.Contig7 | AMQ26131.1 | 99.14 | UDP-glycosyltransferase 25 [Pueraria montana var. lobata] |
| CL2347.Contig2 | KHM98774.1 | 94.34 | Putative glycosyltransferase ypjH [Glycine soja] |
| CL2347.Contig4 | KHM98774.1 | 94.34 | Putative glycosyltransferase ypjH [Glycine soja] |
| CL2424.Contig1 | ADV71370.1 | 98.78 | glycosyltransferase GT14K13 [Pueraria montana var. lobata] |
| CL2424.Contig2 | ADV71370.1 | 98.78 | glycosyltransferase GT14K13 [Pueraria montana var. lobata] |
| CL2424.Contig3 | ADV71370.1 | 98.78 | glycosyltransferase GT14K13 [Pueraria montana var. lobata] |
| CL2846.Contig1 | AGZ84552.1 | 97.94 | glycosyltransferase KGT40 [Pueraria montana var. lobata] |
| CL2846.Contig2 | AMQ26129.1 | 98.75 | UDP-glycosyltransferase 21 [Pueraria montana var. lobata] |
| CL2846.Contig3 | AMQ26129.1 | 98.33 | UDP-glycosyltransferase 21 [Pueraria montana var. lobata] |
| CL2846.Contig4 | ADV71374.1 | 98.19 | glycosyltransferase GT21C20 [Pueraria montana var. lobata] |
| CL2874.Contig1 | XP_003517852.1 | 93.98 | UDP-glycosyltransferase 74B1 [Glycine max] |
| CL3198.Contig2 | RDX67470.1 | 91.5 | Glycosyltransferase-like KOBITO 1 [Mucuna pruriens] |
| CL3364.Contig1 | XP_003535253.1 | 80.11 | probable glycosyltransferase At5g03795 [Glycine max] |
| CL4152.Contig1 | XP_003544901.1 | 90.89 | UDP-glycosyltransferase 87A1 [Glycine max] |
| CL4152.Contig2 | XP_003544901.1 | 91.54 | UDP-glycosyltransferase 87A1 [Glycine max] |
| CL4152.Contig3 | XP_003544901.1 | 91.13 | UDP-glycosyltransferase 87A1 [Glycine max] |
| CL4305.Contig1 | XP_003554404.1 | 90.27 | UDP-glycosyltransferase 73C6 [Glycine max] |
| CL4305.Contig2 | KHN18702.1 | 90.95 | UDP-glycosyltransferase 73C2 [Glycine soja] |
| CL4305.Contig3 | XP_025983683.1 | 90.78 | UDP-glycosyltransferase 73C1 [Glycine max] |
| CL4305.Contig4 | XP_025983683.1 | 86.91 | UDP-glycosyltransferase 73C1 [Glycine max] |
| CL4305.Contig6 | NP_001235380.2 | 92.61 | UDP-glycosyltransferase 73C2 [Glycine soja] |
| CL4395.Contig4 | AMQ26125.1 | 99.37 | UDP-glycosyltransferase 7 [Pueraria montana var. lobata] |
| CL4436.Contig1 | NP_001280039.1 | 89.36 | isoflavone 7-O-glucosyltransferase UGT9 [Glycine max] |
| CL4436.Contig4 | A0A067YBQ3.1 | 89.14 | UDP-glucose:isoflavone 7-O-glucosyltransferase KGT13 [Pueraria montana var. lobata] |
| CL4436.Contig5 | A0A067YBQ3.1 | 89.8 | UDP-glucose:isoflavone 7-O-glucosyltransferase KGT13 [Pueraria montana var. lobata] |
| CL4436.Contig6 | A0A067YBQ3.1 | 88.64 | UDP-glucose:isoflavone 7-O-glucosyltransferase KGT13 [Pueraria montana var. lobata] |
| CL4436.Contig8 | A0A067YB04.1 | 91.93 | UDP-glucose:isoflavone 7-O-glucosyltransferase KGT1 [Pueraria montana var. lobata] |
| CL4614.Contig3 | A0A172J2G3.2 | 99.79 | UDP-glucose:daidzein C-glucosyltransferase [Pueraria montana var. lobata] |
| CL4835.Contig3 | KHN24666.1 | 88.43 | UDP-glycosyltransferase 83A1 [Glycine soja] |
| CL4835.Contig5 | AMQ26126.1 | 96.24 | UDP-glycosyltransferase 10 [Pueraria montana var. lobata] |
| CL5184.Contig1 | XP_003536615.2 | 87.9 | UDP-glycosyltransferase 84B2 [Glycine max] |
| CL5184.Contig2 | XP_003536615.2 | 88.15 | UDP-glycosyltransferase 84B2 [Glycine max] |
| CL5300.Contig2 | XP_003521983.1 | 82.93 | UDP-glycosyltransferase 72D1 [Glycine max] |
| CL5300.Contig3 | XP_003521983.1 | 82.32 | UDP-glycosyltransferase 72D1 [Glycine max] |
| CL5648.Contig1 | ADV71373.1 | 88.03 | glycosyltransferase GT19J14 [Pueraria montana var. lobata] |
| CL5806.Contig3 | XP_003527839.1 | 96.06 | probable glycosyltransferase At5g20260 [Glycine max] |
| CL5806.Contig4 | XP_003527839.1 | 92.78 | probable glycosyltransferase At5g20260 [Glycine max] |
| CL6000.Contig4 | XP_003521422.1 | 88.44 | UDP-glycosyltransferase 73C1 [Glycine max] |
| CL6000.Contig5 | XP_003554403.1 | 89.72 | UDP-glycosyltransferase 73C2 [Glycine max] |
| CL6000.Contig6 | XP_003554403.1 | 86.59 | UDP-glycosyltransferase 73C2 [Glycine max] |
| CL6402.Contig1 | XP_003547777.1 | 82.78 | probable xyloglucan glycosyltransferase 6 [Glycine max] |
| CL6402.Contig2 | XP_003547777.1 | 90.73 | probable xyloglucan glycosyltransferase 6 [Glycine max] |
| CL6402.Contig4 | XP_027359041.1 | 82.83 | xyloglucan glycosyltransferase 4 [Abrus precatorius] |
| CL7048.Contig2 | AMQ26128.1 | 94.66 | UDP-glycosyltransferase 20 [Pueraria montana var. lobata] |
| CL7048.Contig3 | ADV71364.1 | 95.73 | glycosyltransferase GT04F14 [Pueraria montana var. lobata] |
| CL8640.Contig1 | XP_003551309.1 | 87.42 | UDP-glycosyltransferase 91A1 [Glycine max] |
| CL8640.Contig2 | XP_003551309.1 | 87.85 | UDP-glycosyltransferase 91A1 [Glycine max] |
| CL8640.Contig3 | XP_003552084.1 | 87.31 | UDP-glycosyltransferase 91A1 [Glycine max] |
| CL8660.Contig10 | AGZ84549.1 | 93.66 | glycosyltransferase KGT31 [Pueraria montana var. lobata] |
| CL8660.Contig1 | AGZ84549.1 | 91.09 | glycosyltransferase KGT31 [Pueraria montana var. lobata] |
| CL8660.Contig2 | AGZ84549.1 | 96.84 | glycosyltransferase KGT31 [Pueraria montana var. lobata] |
| CL8660.Contig3 | AGZ84549.1 | 96.84 | glycosyltransferase KGT31 [Pueraria montana var. lobata] |
| CL8660.Contig5 | AGZ84549.1 | 91.09 | glycosyltransferase KGT31 [Pueraria montana var. lobata] |
| CL8660.Contig6 | AGZ84549.1 | 91.09 | glycosyltransferase KGT31 [Pueraria montana var. lobata] |
| CL8660.Contig9 | AGZ84549.1 | 88.07 | glycosyltransferase KGT31 [Pueraria montana var. lobata] |
| CL8777.Contig2 | XP_020227670.1 | 93.96 | probable galactinol--sucrose galactosyltransferase 1 [Cajanus cajan] |
| CL9305.Contig1 | XP_003531850.1 | 83.66 | UDP-glycosyltransferase 83A1 [Glycine max] |
| CL9612.Contig1 | XP_003550913.1 | 84.1 | probable glycosyltransferase At5g03795 [Glycine max] |
| CL9612.Contig2 | XP_003550913.1 | 89.95 | probable glycosyltransferase At5g03795 [Glycine max] |
| CL9616.Contig1 | XP_003535921.1 | 95.39 | probable glycosyltransferase STELLO2 [Glycine max] |
| CL9616.Contig2 | XP_020207432.1 | 95.54 | probable glycosyltransferase STELLO1 [Cajanus cajan] |
| CL9618.Contig2 | NP_001240903.1 | 87.63 | UDP-glycosyltransferase 71D1-like [Glycine max] |
| CL9618.Contig3 | NP_001240903.1 | 87 | UDP-glycosyltransferase 71D1-like [Glycine max] |
| CL9618.Contig4 | XP_003519247.1 | 87.31 | UDP-glycosyltransferase 71K2 [Glycine max] |
| Unigene11684 | AMQ26127.1 | 99.39 | UDP-glycosyltransferase 19 [Pueraria montana var. lobata] |
| Unigene11714 | XP_003543833.1 | 96.69 | xyloglucan glycosyltransferase 4 [Glycine max] |
| Unigene12699 | XP_027359181.1 | 91.33 | glycosyltransferase BC10-like [Abrus precatorius] |
| Unigene13648 | XP_003554405.1 | 87.86 | UDP-glycosyltransferase 73C3 [Glycine max] |
| Unigene1400 | XP_003554601.1 | 91.31 | UDP-glycosyltransferase 74G1 [Glycine max] |
| Unigene14239 | AMQ26133.1 | 99.57 | UDP-glycosyltransferase 3 [Pueraria montana var. lobata] |
| Unigene14388 | XP_003556840.1 | 91.16 | UDP-glycosyltransferase 85A2 [Glycine soja] |
| Unigene14756 | XP_020219871.1 | 74.61 | UDP-glycosyltransferase 83A1-like [Cajanus cajan] |
| Unigene15238 | XP_003548705.1 | 93.94 | Putative glycosyltransferase [Glycine soja] |
| Unigene18616 | XP_003521002.1 | 87.18 | UDP-glycosyltransferase 72B1 [Glycine soja] |
| Unigene18707 | XP_003544918.1 | 77.85 | UDP-glycosyltransferase 82A1 [Glycine max] |
| Unigene20293 | AGZ84548.1 | 98.65 | glycosyltransferase KGT22 [Pueraria montana var. lobata] |
| Unigene21048 | XP_006573464.1 | 84.65 | probable glycosyltransferase At3g07620 [Glycine max] |
| Unigene21841 | AGZ84547.1 | 98.68 | glycosyltransferase KGT18 [Pueraria montana var. lobata] |
| Unigene2584 | XP_018808849.1 | 55.15 | UDP-glycosyltransferase 88B1-like [Juglans regia] |
| Unigene2641 | KHN41660.1 | 94.14 | Putative glycosyltransferase [Glycine soja] |
| Unigene2846 | XP_014630244.1 | 93.25 | Putative glycosyltransferase [Glycine soja] |
| Unigene3016 | XP_003535222.1 | 88.48 | UDP-glycosyltransferase 73C3 [Glycine max] |
| Unigene3560 | XP_014618506.1 | 92.59 | UDP-glycosyltransferase 73D1 [Glycine max] |
| Unigene362 | XP_003548145.1 | 88.7 | UDP-glycosyltransferase 88A1 [Glycine max] |
| Unigene3776 | AMQ26118.1 | 96.88 | UDP-glycosyltransferase 48 [Pueraria montana var. lobata] |
| Unigene383 | AMQ26119.1 | 99.38 | UDP-glycosyltransferase 42 [Pueraria montana var. lobata] |
| Unigene54337 | AGZ84548.1 | 95.94 | glycosyltransferase KGT22 [Pueraria montana var. lobata] |
| Unigene54338 | AGZ84548.1 | 89.39 | glycosyltransferase KGT22 [Pueraria montana var. lobata] |
| Unigene54862 | A0A067YB04.1 | 98.75 | UDP-glucose:isoflavone 7-O-glucosyltransferase KGT1 [Pueraria montana var. lobata] |
| Unigene55419 | KHN13565.1 | 86.83 | UDP-glycosyltransferase 83A1 [Glycine soja] |
| Unigene5981 | XP_027359181.1 | 91.33 | glycosyltransferase BC10-like [Abrus precatorius] |
| Unigene67442 | AMQ26120.1 | 99.15 | UDP-glycosyltransferase 45 [Pueraria montana var. lobata] |
| Unigene7178 | XP_027359181.1 | 91.33 | glycosyltransferase BC10-like [Abrus precatorius] |
| Unigene7847 | XP_003539219.1 | 92.45 | glycosyltransferase-like KOBITO 1 [Glycine max] |
| Unigene8324 | ADV71361.1 | 99.18 | glycosyltransferase GT02J01 [Pueraria montana var. lobata] |
| Unigene9715 | XP_003550126.1 | 89.95 | probable glycosyltransferase At3g07620 [Glycine max] |
| CL4436.Contig9 | AMQ26113.1 | 98.09 | UDP-glucose:isoflavone 7-O-glucosyltransferase [Pueraria montana var. lobata] |
